# Ellis-van Creveld Syndrome: A Rare Clinical Report of Oral Rehabilitation by Interdisciplinary Approach

**DOI:** 10.1155/2018/8631602

**Published:** 2018-01-23

**Authors:** Talib Amin Naqash, Ibrahim Alshahrani, Siripan Simasetha

**Affiliations:** ^1^Department of Prosthetic Dentistry, King Khalid University College of Dentistry, Abha, Saudi Arabia; ^2^Department of Pediatric Dentistry and Orthodontic Sciences, King Khalid University College of Dentistry, Abha, Saudi Arabia; ^3^Dental Department, Bhumibol Adulyadej Hospital, The Royal Thai Air Force, Bangkok, Thailand

## Abstract

Ellis-van Creveld syndrome (EVC) is a very rare genetic disorder that affects various tissues of ectodermal and mesodermal origin; patients with EVC present with typical oral deficiencies. The affected individuals are quite young at the time of oral evaluation. It is, therefore, important that these individuals are diagnosed and receive dental treatment at an early age for their physiologic and psychosocial well-being. Albeit there are numerous articles penned on the EVC, the treatise from an oral perspective is inadequate, covering only oral exhibitions and the preventive treatments. This article reviews the literature and serves as the first disquisition for oral rehabilitation of an EVC patient utilizing surgical, orthodontic, restorative, and prosthodontic management.

## 1. Introduction

Ellis-van Creveld syndrome (EVC) is a rare autosomal recessive disorder with characteristic clinical manifestations, resulting from a genetic mutation in two genes, *EVC1* and *EVC2*, mapping both in locus 16 on the short arm of chromosome 4 (4p16) in a head-to-head configuration [1, 2]. EVC presents with a distinctive tetrad of disproportionate dwarfism, bilateral postaxial polydactyly, ectodermal dysplasia, and congenital heart malformations [3]. It is also known as chondroectodermal dysplasia and mesoectodermal dysplasia; dysplasia is an abnormality in form or development [4].

Pediatricians Richard W. B. Ellis of Edinburgh and Simon van Creveld of Amsterdam were the first to describe a case of EVC in 1940 [5]. The syndrome had been partially described previously in several reports, but work of Ellis and van Creveld defined it [6, 7]. In literature, detailed description of clinical presentation in finite case series or single reports is found [3–13].

EVC presents with a characteristic tetrad of clinical manifestations [3]:

1. Chondrodysplasia of the long, tubular bones resulting in disproportionate dwarfism, and an exceptionally long trunk is the most common clinical feature, producing a serious ossification defect [6]. The severity of short limbs increases from the proximal to the distal portions [6].
2. Bilateral postaxial polydactyly of the hands, with the supernumerary finger, usually being on the ulnar side [6]. Fingers are sausage shaped with wide hands and feet [14].
3. Hidrotic ectodermal dysplasia with dystrophic, small dysplastic nails, thin sparse hair, and oral manifestations [12].
4. Congenital heart malformations in 50% to 60% of cases, the most common being a single atrium and a ventricular septal defect [6]. The associated cardiorespiratory problems are described as the primary cause of decreased life expectancy in these patients [15].

According to Winter and Geddes, oral manifestations in EVC are characteristic and remarkable [16]. The most common finding is the fusion of the anterior portion of the upper lip to the maxillary gingival margin, obliterating mucolabial fold, causing the upper lip to present a slightly V-notch in the middle [14, 17]. The anterior portion of the lower alveolar ridge is often jagged [7]. Multiple small accessory labiogingival frenula, serrated incisal edges, diastemas, teeth of abnormal form, enamel hypoplasia, and hypodontia are other features [3, 16]. Varela and Ramos stated that malocclusion is secondary to oral abnormalities and is of no specific type [18].

## 2. Clinical Report

**Figure 1:**
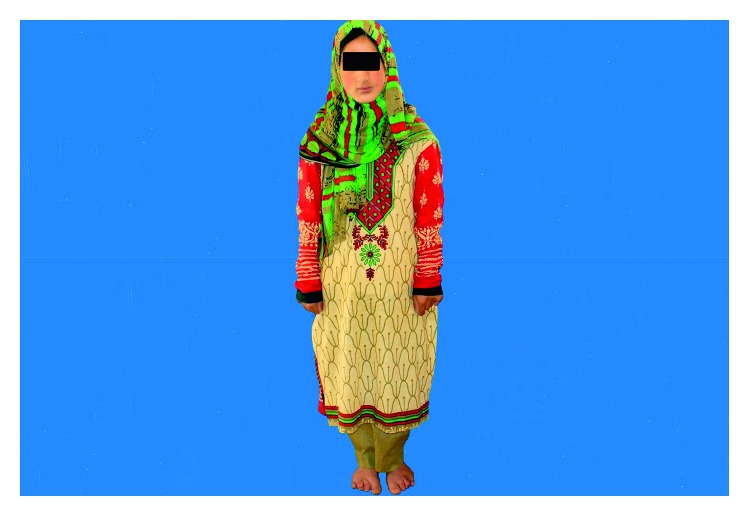
Patient with Ellis-van Creveld syndrome.

A 15-year-old female was referred to the Department of Prosthetic Dentistry for evaluation and prosthetic dental treatment of congenitally absent maxillary lateral incisors and mandibular incisors (Figure 1). The patient was attending a regular school but had concerns about her esthetics.

**Figure 2:**
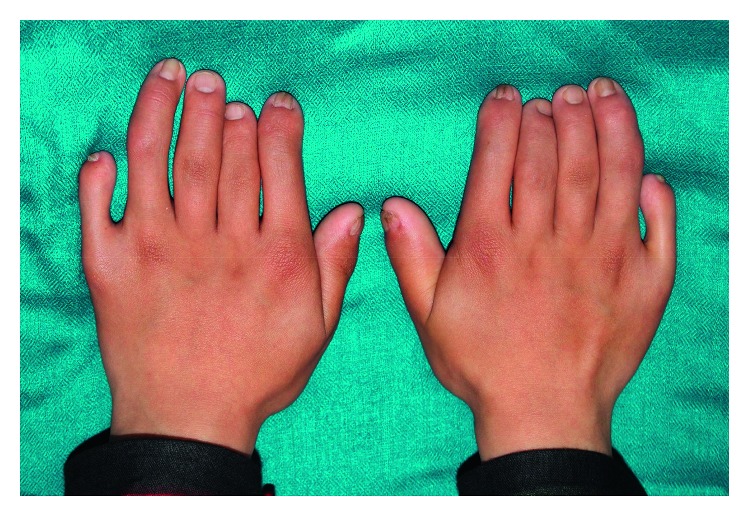
Hands showing polydactyly and hypoplastic nails.

Pregnancy and delivery were uneventful, and no exposure to radiation or drugs had occurred during pregnancy. At birth, however, the patient presented with short limbs, a long trunk, and polydactyly of hands. Medical history revealed that the patient has an atrial septal defect and was being planned for surgical closure. Psychomotor development was within the normal range. Extra oral examination showed that the patient has short limb dwarfism (131 cm), with a long trunk and weighed 37.1 kg. Polydactyly of hands was observed with dysplastic and atrophic finger and toe nails (Figure 2). Hair was thin and sparse.

**Figure 3:**
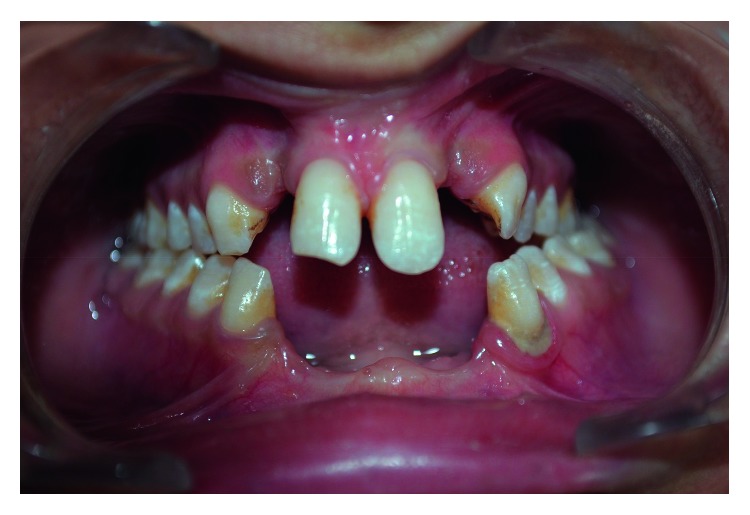
Absent maxillary lateral incisors and mandibular incisors, large maxillary labial frenum, multiple accessory labial frenula, midline diastema, mandibular anterior ridge defect, and crossbite.

Intraoral examination showed absence of maxillary lateral incisors, mandibular central and lateral incisors, microdontia of the maxillary left canine, unilateral crossbite on the left side, partial end-to-end occlusal relationship on the right side, and alveolar ridge defect in the anterior mandible (Figure 3).

The examination of soft tissues showed presence of a large maxillary labial frenum attached to alveolar ridge causing obliteration of vestibule and midline diastema. Laterally, there were multiple small accessory labial frenula (Figure 3). The remaining oral mucosa was normal.

**Figure 4:**
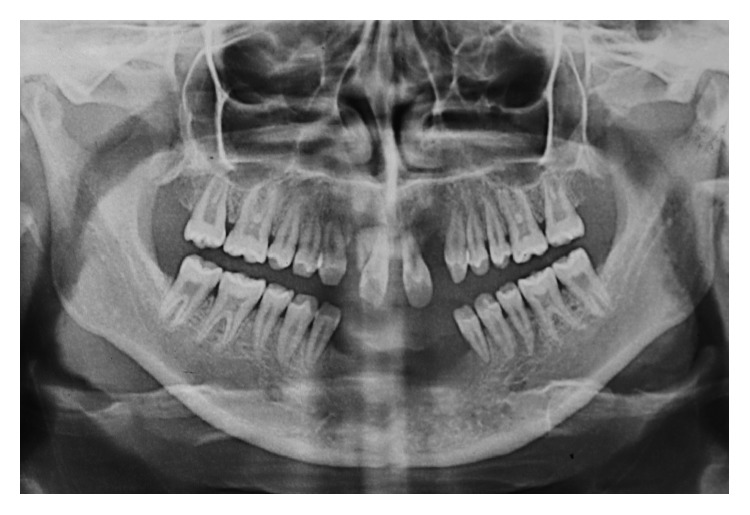
Panoramic radiograph showing agenesis of maxillary lateral incisors, mandibular incisors, and all 4 third molars.

A panoramic radiograph confirmed agenesis of the maxillary lateral incisors, mandibular incisors, and all third molars (Figure 4).

Dental procedure that involved manipulation of gingival tissue or perforation of the oral mucosa was performed under proper antibiotic cover, as per revised guidelines from American Heart Association, to prevent infective (bacterial) endocarditis [19].

Treatment started with supragingival periodontal therapy for removal of plaque and calculus, and to improve oral health. It was followed by maxillary labial frenectomy and vestibular deepening, using electrosurgery. Electrocautery procedure offered minimal time consumption, bloodless field during the surgical procedure with no requirement of sutures, and absence of postoperative complications [20].

**Figure 5:**
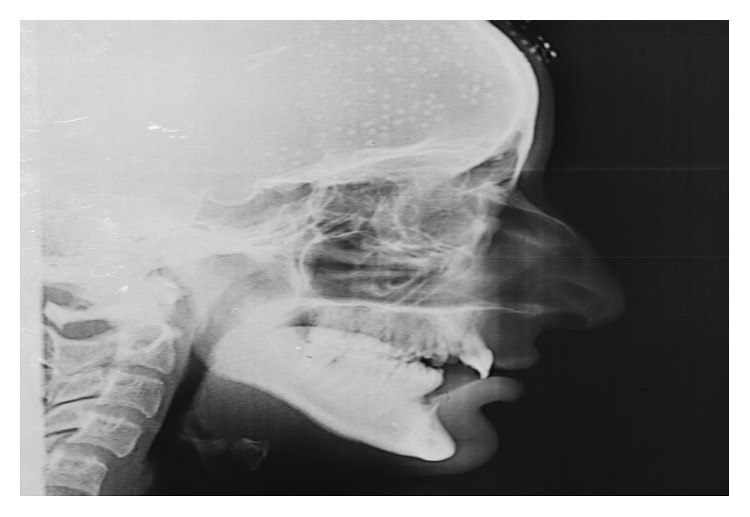
Lateral cephalogram.

**Figure 6:**
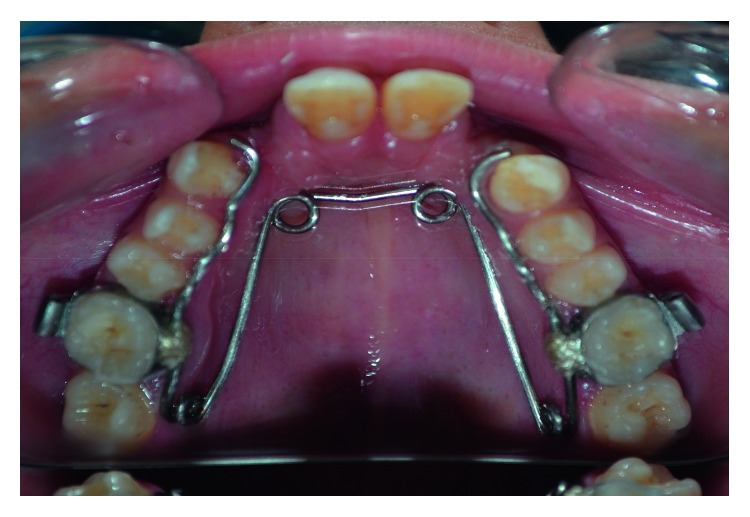
Quad helix cemented to maxillary molars.

Following postoperative healing, orthodontic examination revealed that the patient had a unilateral crossbite on the left side. Cervical Vertebrae Maturation Index (CVMI), using lateral cephalogram (Figure 5), depicted the patient to be in CVMI Stage V [21, 22]. As such, the patient was put on Quad Helix, a slow maxillary expansion appliance, aimed at dentoalveolar expansion of the arch on the left side and correction of partial end-to-end occlusion on the right side [23, 24]. The appliance was fabricated from 36 mil stainless steel wire and was soldered with bands. Initial activation of 8 mm was done extraorally, and the bands were cemented with glass ionomer cement (Ketac Cem Glass Ionomer Cement, 3M) on maxillary molars (Figure 6). The patient was seen every four weeks for three months unless the appliance achieved 8 mm intraoral activation. After twelve-week treatment, crossbite was corrected and the appliance was removed. A retention appliance was placed for three months to prevent relapse.

After correction of crossbite, crown build-up, with glass ionomer cement (Vitremer, 3M), was done on the maxillary left canine, to correct microdontia.

**Figure 7:**
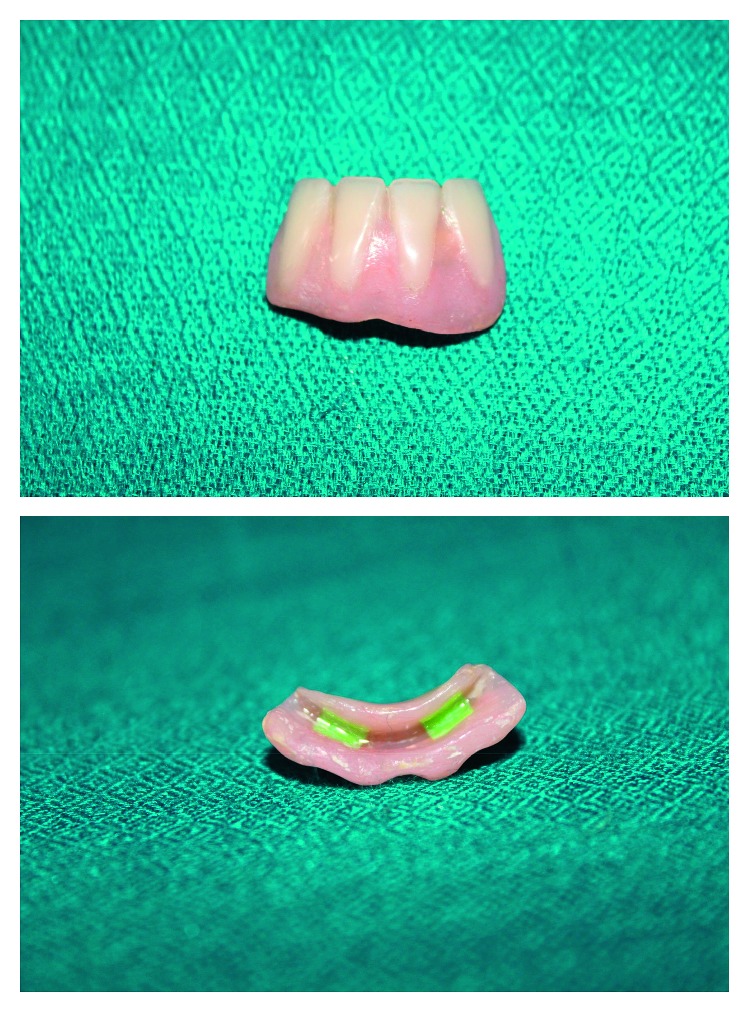
Andrew's removable component with Ceka Preciline attachments.

Andrew's Bridge System was designed for rehabilitation of mandibular incisors on lower canines, keeping in view Seibert's class III ridge defect in the anterior mandible [25]. Andrew's Bridge System is a fixed removable prosthesis that is indicated in patients with large ridge defects. It provides maximum aesthetics, is hygienic, and has a good fit with minimal trauma to soft tissues or underlying bone at an economic price [26, 27]. Bar and Clip attachments (Preci-Horix, Ceka) were used to secure removable and fixed component (Figure 7).

Dental implants were planned for maxillary lateral incisors, but the patient was reluctant to undergo invasive treatment option, owing to her concerns about the cardiac defect. Therefore, six units metal ceramic fixed dental prosthesis (Ivoclar Vivadent) was fabricated in the maxillary arch, from canine to canine region, with maxillary canines and central incisors as abutments.

The patient was trained to properly insert and remove the removable prosthesis that was fabricated over the fixed component of Andrew's Bridge System, and proper oral hygiene instructions (including interdental brush) were given to the patient.

Follow-up was done for six months, and no complication after treatment was noted.

## 3. Discussion

The presentation of medically compromised and syndromic children in the dental office is a great challenge to oral health care providers [28]. Various syndromes are identified earlier in childhood and demand special attention right from the birth [28]. EVC is one of these syndromes with variable phenotype affecting multiple organs [11].

There is no definitive cure for EVC [29]. The management is multidisciplinary which involves several specialists: a cardiologist, a pediatrician, an orthopedician, a prosthodontist, an oral and maxillofacial surgeon, an orthodontist, and a periodontist [13, 30].

The approach to dental management will depend on each particular case [6]. Preventive measures include dietary counseling, plaque control, oral hygiene instructions, fluoride varnish application, or daily fluoride mouth rinses [3, 6, 31].

To maintain space and to improve function, esthetics, and speech, removable or fixed dental prosthesis (considering age) is recommended [28]. Restoration of hypoplastic and decayed teeth is indicated to preserve tooth structure and to improve esthetics; taking into account possible presence of enlarged pulp chambers [6, 30]. For soft tissue anomalies, surgical correction is advised [31]. Parental and child counseling is often required to treat psychological trauma due to compromised oral and medical health [28].

## 4. Summary

EVC is a rare autosomal recessive disorder with variable expression, diagnosed by its characteristic clinical manifestations. Dental and oral manifestations of EVC are definitive; dentist plays a vital role in its early diagnosis and treatment planning and to establish a differential diagnosis with other clinically similar entities. EVC has high mortality in early life due to cardiac and respiratory problems; those who survive require multidisciplinary treatment planning in terms of preventing oral diseases and providing rehabilitation. Early treatment can help the patient to prevent various problems and undue psychological trauma.

**Figure 8:**
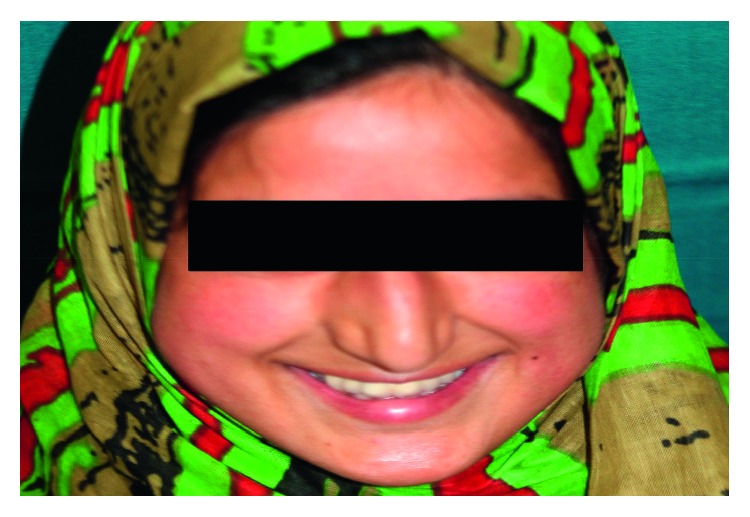
Clinical view after oral rehabilitation.

After completion of the treatment, esthetics, function and phonetics improved remarkably. The patient was happy and comfortable with the oral rehabilitation, and the post treatment esthetic outcome helped her to improve her quality of life (Figure 8).
